# Generation of *Myostatin* Gene-Edited Channel Catfish (*Ictalurus punctatus*) via Zygote Injection of CRISPR/Cas9 System

**DOI:** 10.1038/s41598-017-07223-7

**Published:** 2017-08-04

**Authors:** Karim Khalil, Medhat Elayat, Elsayed Khalifa, Samer Daghash, Ahmed Elaswad, Michael Miller, Hisham Abdelrahman, Zhi Ye, Ramjie Odin, David Drescher, Khoi Vo, Kamal Gosh, William Bugg, Dalton Robinson, Rex Dunham

**Affiliations:** 1 School of Fisheries, Aquaculture and Aquatic Sciences, Auburn University, Auburn, AL 36849 USA; 2 Anatomy and Embryology Department, Faculty of Veterinary Medicine, Cairo University, Giza, 12211 Egypt; 3 Harrison School of Pharmacy, Auburn University, Auburn, AL 36849 USA; 4 Department of Animal Wealth Development, Faculty of Veterinary Medicine, Suez Canal University, Ismailia, 41522 Egypt; 5 Department of Veterinary Hygiene and Management, Faculty of Veterinary Medicine, Cairo University, Giza, 12211 Egypt

## Abstract

The myostatin (MSTN) gene is important because of its role in regulation of skeletal muscle growth in all vertebrates. In this study, CRISPR/Cas9 was utilized to successfully target the channel catfish, *Ictalurus punctatus*, muscle suppressor gene MSTN. CRISPR/Cas9 induced high rates (88–100%) of mutagenesis in the target protein-encoding sites of MSTN. MSTN-edited fry had more muscle cells (*p* < 0.001) than controls, and the mean body weight of gene-edited fry increased by 29.7%. The nucleic acid alignment of the mutated sequences against the wild-type sequence revealed multiple insertions and deletions. These results demonstrate that CRISPR/Cas9 is a highly efficient tool for editing the channel catfish genome, and opens ways for facilitating channel catfish genetic enhancement and functional genomics. This approach may produce growth-enhanced channel catfish and increase productivity.

## Introduction

Although fish comprise the most diverse group among the vertebrates, a much smaller number of fish species is used for research and commercial purposes. The major species cultured in the world include salmonids, tilapias, carps and catfishes. There are about 40 extant families, and more than 3,700 extant species of catfishes that have been identified and described, living in marine and fresh waters^1^. This makes the Siluriformes the second most diverse vertebrate order; in fact, one of every twenty vertebrate species is a catfish. Members of the family *Ictaluridae*, especially the genus *Ictalurus* and their hybrids, are the primary fish species used in aquaculture and sport fishing in the United States. Recently, the production and the water surface area devoted to catfish culture in the United States declined^2^ due to several factors, including increasing fuel and feed cost and competition from foreign imports. Genetic improvement of catfish offers a promising approach for this industry to maintain profitability and sustainability via development of high performance fish^3^.

Myostatin (MSTN), or growth and differentiation factor 8, is one of the well-known examples of economically important genes. Mutations linked with the double-muscled phenotype in Belgian Blue and Piedmontese cattle were found in the MSTN gene, resulting in a significant increase in muscling compared to conventional cattle^4–7^. MSTN was identified as a member of the transforming growth factor *β* (TGF-*β*) superfamily that initiate their roles in regulation of skeletal muscle mass through the cell surface receptor, activin receptors type II (ACVR2)^8–10^. Myostatin is a key regulator of skeletal muscle growth in all vertebrates and regulates myoblast differentiation *in vitro* ^11^. Altering myostatin, through gene knockout^12^ or overexpression of inhibitors^9^, prominently increases muscle mass. Mutations of myostatin were also found in other cattle breeds^13, 14^ and mammalian species^15–18^. Whereas the mammalian myostatin is exclusively expressed from one gene copy and limited to skeletal muscle^6, 12, 19^, two or four copies have been found in fishes^20–26^ and differentially expressed in many tissues^27–36^ such as muscle, eye, stomach, skin, brain, gonads, kidney, intestine, liver, spleen, gill and heart. In channel catfish (*Ictalurus punctatus*), the MSTN protein is actively expressed only from one copy on chromosome 6 in different tissues^30, 37^. The MSTN protein is conserved in teleosts, but its functions in fish remain to be revealed because of the lack of spontaneous MSTN null mutation. The knockout of MSTN would further the study on the roles of this gene in fish species.

Different molecular editing tools have been used to target MSTN in teleosts, such as zinc-finger nucleases (ZFNs)^38^ and transcription activator-like effector nucleases (TALENs)^39, 40^. Although the use of such engineered nucleases made the production of gene-edited animals possible, they are difficult to engineer and showed low specificity to target DNA sequences^41^. Recently, a new gene editing system has been applied with higher targeting efficiency and lower cell toxicity, known as clustered regularly interspaced short palindromic repeats (CRISPR)/CRISPR-associated protein 9 (Cas9). Based on the type II prokaryotic CRISPR from *Streptococcus pyogenes* ^42, 43^, the co-delivery of endonuclease Cas9 combined with a synthetic small guide RNA (sgRNA) targeting certain gene(s) into eukaryotic cells can edit the genome by stimulating a double-strand break (DSB) at a desired site(s). Then the non-homologous end-joining (NHEJ) process re-ligates the DSBs, generating insertion/deletion (indel) mutations and enabling existing genes to be edited, deleted or new ones inserted. Unlike ZFNs and TALENs, sgRNA is the only component that needs designation for each genomic target, thereby significantly simplifying the design and lowering the cost of gene editing compared to the protein-based target recognition platforms. To date, CRISPR/Cas9 has been applied extensively in many species and promises to transform the fields of genome editing in plants and animals. MSTN knockout strains utilizing zygote microinjection of CRISPR/Cas9 have been produced recently for laboratory animals^44–46^ and livestock^47–50^. Multiplex genome editing using Cas9 endonuclease also can be applied easily by co-delivering a combination of sgRNAs to target multiple genomic loci simultaneously^51–54^. Recently, CRISPR mutagenesis was performed to edit various genes (including MSTN) in some aquaculture species^55–59^.

**Figure 1:**
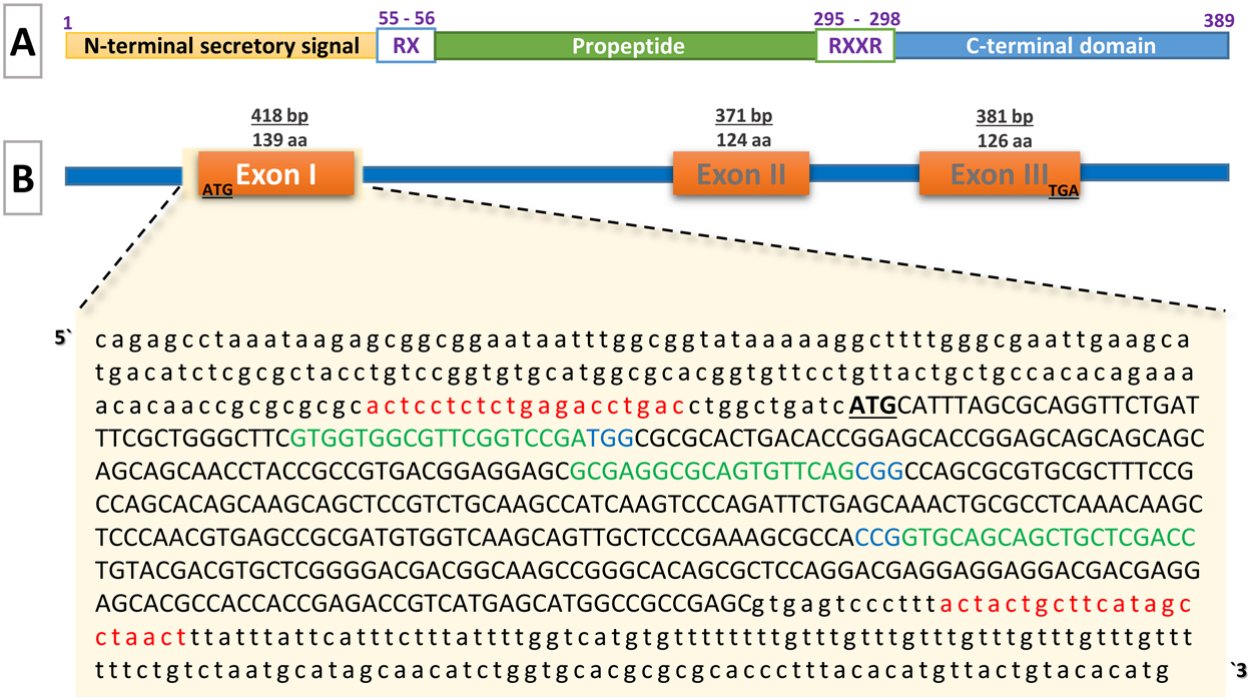
Design of the CRISPR/Cas9 system. (**A**) Schematic diagram of channel catfish MSTN; a signal sequence (N-terminal secretory signal), a propeptide domain (propeptide) and a bioactive domain (C-terminal domain). RX (55–56) is a proteolytic site to remove the signal sequence and RXXR (295–298) is a proteolytic processing site (RSSR) to produce C- terminal bioactive form of MSTN. These 3 domains (389 amino acids) are encoded from (**B**) the three exons of the channel catfish MSTN gene; Exons I, II and III consist of 418, 371 and 381 base-pairs encoding 139, 124 and 129 amino acids residues, respectively. CRISPR/Cas9 target sites in Exon I; The Exon and Introns are indicated by upper and lower case, the underlined Bold uppercase are the start and stop codons, The primers used in PCR are indicated in red, The guide RNA target sites are indicated in green followed by PAM (Protospacer adjacent motif, NGG) in Blue.

The channel catfish MSTN gene has been cloned and characterized^30, 37^, and has only one orthologous MSTN gene in their genome. Like other MSTN genes in mammals and teleost, the *I. punctatus* MSTN gene consists of three exons and two introns, and encodes a protein consisting of 389 amino acids that span three conserved domains with two proteolytic sites in between dibasic (RX) and tetrabasic (RXXR) at amino acids (55–56) and (295–298), respectively (Fig. 1A). The roles of myostatin in channel catfish, however, still need further investigation. In this study, we used CRISPR zygote microinjection to knockout MSTN gene in channel catfish, and determined the effects of knockout on growth. Due to the long generation time of channel catfish, we limited our study to early growth. We aim to produce growth-enhanced lines of channel catfish with CRISPR technology. These gene-edited lines would then be fully characterized for growth rate, feed efficiency and disease resistance and compared to genotypes currently used in aquaculture.

## Results

### Design and Delivery of CRISPR/Cas9 System

CRISPRscan online tool was used to design oligos to generate MSTN edited channel catfish. Three guide RNAs were designed targeting three different MSTN sites that were followed by the PAM (**P**rotospacer **A**djacent **M**otif, NGG) sequence along exon I to alter the expression of the channel catfish MSTN gene (Fig. 1B) (Table 1). The designed sgRNA(s) were co-injected with Cas9 nuclease individually and also mixed with the other sgRNAs, resulting in four groups, MSTN-1, MSTN-2, MSTN-3 and MSTN-Mix (a combination of -1, -2 and -3). Additionally, two control groups were included; one a buffer-injected control without any sgRNA (iCTRL), and the other a normal non-injected control (nCTRL). For each treatment group, embryo mortality, hatchability, fry survival and mutation rate were recorded and compared statistically (Table 2).

**Table 1:** The sequences of small guide RNAs and the universal (common) primer used to target exon I of the channel catfish myostatin (MSTN) gene.

guide RNA ID	Oligo sequence* (5′-3′)	Locus on strand	CRISPR scan score %
MSTN-1	taatacgactcactata**GGTGGTGGCGTTCGGTCCGA**gttttagagctagaa	+	81
MSTN-2	taatacgactcactata**GGGCGAGGCGCAGTGTTCAG**gttttagagctagaa	+	75
MSTN-3	taatacgactcactata**GGGTCGAGCAGCTGCTGCAC**gttttagagctagaa	−	70
Universal primer	aaaagcaccgactcggtgccactttttcaagttgataacggactagccttattttaacttgctatttctagctctaaaac		

*The bold uppercase letters designate the target sequences (see Fig. 1).

**Table 2:** The survival and hatchability of embryos, fry survival and mutation rate of the channel catfish embryos microinjected at the one-cell stage with sgRNAs/Cas9 protein targeting the myostatin (MSTN) gene.

Treatment	Embryos injected	Embryo mean time to death (days) ± *SEM*	Live embryos and Hatch		Mean time to hatch (days) ± *SEM*	Fry survival	Fry mean time to death (days) ± *SEM*	Mutated dead embryos	Mutated hatched fry
	*N*		*N*	%		%		%	%
MSTN-1	82	6.1 ± 0.30^a^	44	53.7	7.2 ± 0.08^b^	90.9	19.0 ± 0.05	100	88.6
MSTN-2	28	4.6 ± 0.46^b^	7	25.0	7.0 ± 0.00^ab^	100	No mortality	100	100
MSTN-3	88	6.3 ± 0.22^a^	50	56.8	6.9 ± 0.04^a^	84.0	18.3 ± 0.58	100	88.0
MSTN-Mix	82	5.2 ± 0.27^b^	27	32.9	7.2 ± 0.08^b^	85.2	18.3 ± 0.81	100	96.3
iCTRL	137	6.2 ± 0.20^a^	83	60.6	7.1 ± 0.04^b^	95.2	19.6 ± 0.24	—	—
nCTRL	284	7.6 ± 0.08	220	77.5	7.4 ± 0.04	93.6	20.0 ± 0.03	—	—

Three sgRNAs were microinjected individually (MSTN-1, MSTN-2 and MSTN-3) and multiplexed (MSTN-Mix). Two controls were used; injected control embryos (iCTRL) were full-siblings to the treatment groups and were injected with the same solution and volume, but without sgRNA or Cas9 protein. The second control was not injected (nCTRL). Hatch % is the number of live embryos in each treatment divided by the total number of embryos in the same treatment and multiplied by 100. Pairwise comparisons of mean survival and hatch time were performed using Log Rank (Mantel-Cox) test with SPSS 23.0 software. All data are presented as the mean ± standard error (*SEM*). Means followed by different superscript letters are significantly different (*p* < 0.05). Mutation rates are calculated based on Surveyor analysis of all dead embryos and hatched fry.

### Embryo Mortality, Hatchability, Fry Survival Rates

#### Embryo mortality

**Figure 2:**
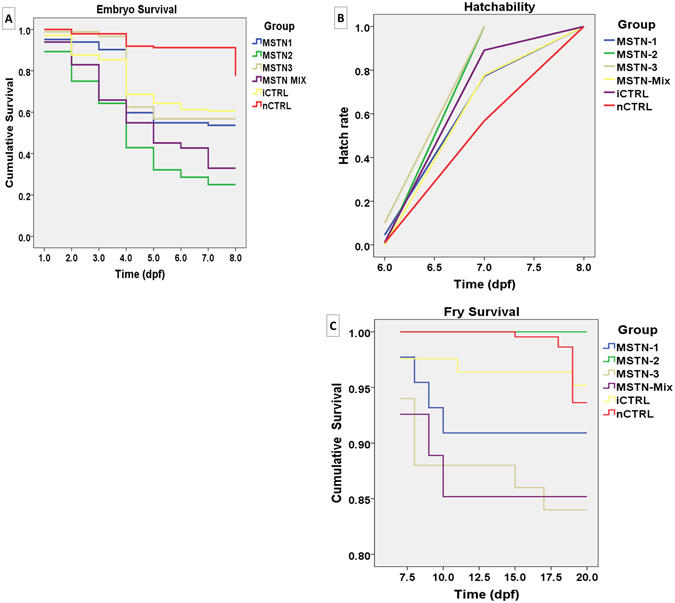
Plots of (**A**) embryo survival, (**B**) mean time to hatch, and (**C**) fry survival; embryos microinjected at the one-cell stage with sgRNAs/Cas9 protein targeting the channel catfish MSTN gene. Three sgRNAs were microinjected individually (MSTN-1, MSTN-2 and MSTN-3) and multiplexed (MSTN-Mix). Two controls were used; Injected control embryos (iCTRL) were full-siblings to the treatment groups and were injected with the same solution and volume but without sgRNA or Cas9 protein. The second control was not injected (nCTRL). Hatch rate was calculated as the number of embryos that hatched at a given time-point (day post-fertilization, dpf) compared to the total number of embryos that hatched.

The mortality began at the first day post-fertilization (dpf) and continued until eight dpf. Embryo mortality was lowest in nCTRL embryos, followed by iCTRL embryos (Fig. 2A). Mean survival time ranged from 4.6 dpf in MSTN-2 group to 7.6 dpf in nCTRL group. Overall comparisons revealed significant differences in the mean survival time among at least two groups (*p* < 0.0001). With pairwise comparisons, the mean survival time for the nCTRL group was significantly longer than for all other groups (*p* < 0.0001). Mean survival time was different for the following groups (*p* < 0.005): MSTN-1 and MSTN-2, MSTN-1 and MSTN-Mix, MSTN-2 and MSTN-3, MSTN-2 and iCTRL, MSTN-Mix and iCTRL, and iCTRL and nCTRL. All other pairwise comparisons of mean survival time were not significantly different (*p* > 0.05) (Table 2).

#### Embryo hatch

Embryos began to hatch at six dpf, and hatch was completed at eight dpf. Embryo mean time to hatch ranged from 6.9 dpf in MSTN-3 group to 7.4 dpf in nCTRL group (Fig. 2B, Table 2). Overall comparison of embryo mean time to hatch revealed significant differences among at least two groups (*p* < 0.0001). The nCTRL group had the longest hatch time when compared to all other groups (*p* < 0.05). MSTN-3 group hatched earlier than the MSTN-1 (*p* = 0.001), MSTN-Mix (*p* = 0.0003) and iCTRL (*p* = 0.001) groups. All other pairwise comparisons of mean time to hatch were not different (*p* > 0.05). Fry mean survival time ranged from 18.3 to 20.0 dpf (Fig. 2C). No significant differences in fry survival time were detected among all groups (*p* = 0.078) (Table 2). No deformed or abnormal fry were seen in any treatments both pre- and post-hatch.

### Analysis of Mutagenesis Efficiencies of CRISPR/ Cas9

**Figure 3:**
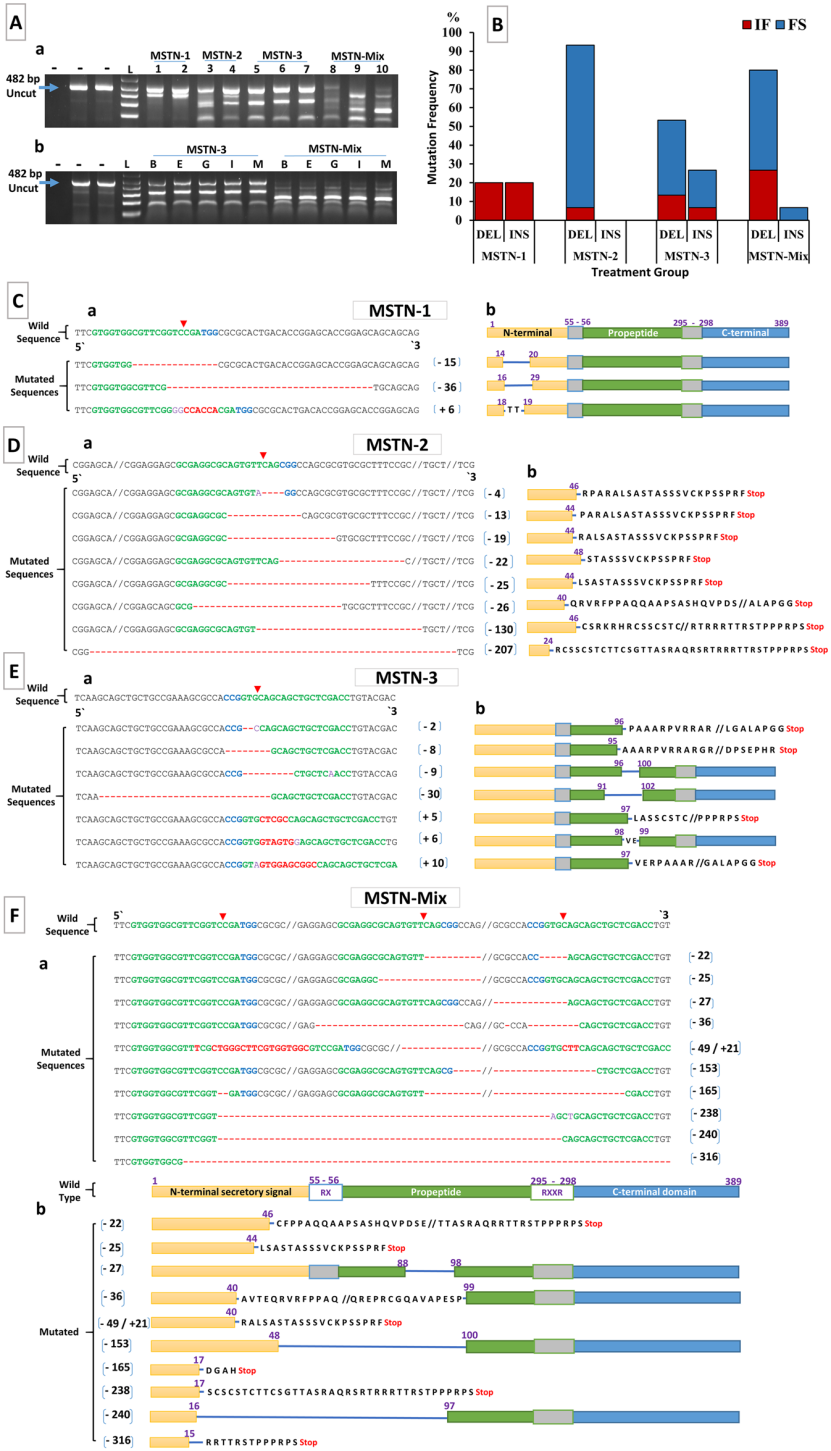
Analysis of mutagenesis efficiencies of CRISPR/ Cas9. (**A**) Identification of edited myostatin (MSTN) gene sequences in channel catfish using the surveyor mutation detection assay. Minus sign indicates the negative control without template [first lane] and the control with wild-type DNA as template [2^nd^ and 3^rd^ lanes] (482 bp). L indicates 1 kb plus DNA ladder (Invitrogen, Carlsbad, CA). (a) surveyor mutation detection of MSTN gene in fin-clip samples from 10 individuals from different treatments. Two or more bands indicate that mutations occurred ( < 482 bp). (b) surveyor mutation detection of MSTN gene in different tissues from two mutated channel catfish: B Barbel; E Eye; G Gills; I Intestine; M Muscle. Electrophoretic results were cropped from the original images shown in Supplementary Fig. S1. (**B**) Insertion (INS) and deletion (DEL) mutation frequencies in the treatment groups with in-frame (IF) and frame-shift (FS) mutation percentages. (**C–F**) CRISPR-Cas9-induced mutagenesis efficiencies for the MSTN gene in different treatment groups; (**C**) MSTN-1 (**D**) MSTN-2 (**E**) MSTN-3 (**F**) MSTN-Mix. (a) Sequences of channel catfish MSTN gene with co-delivered sgRNA(s) and Cas9 protein induced mutations. The wild-type channel catfish MSTN gene sequences are shown on the top. Sequences in green are the target sites of guide RNA followed by PAM sequence (Blue); Red arrows indicate the expected sites of cleavage by Cas9. Red dashes and letters indicate the deletion/insertion of nucleotides. Numbers in brackets shows the number of nucleotides deleted (−) or inserted (+) in the edited MSTN gene. (b) Schematic diagrams show predicted truncated proteins that would be produced from the mutated catfish (see **C–Fa**). The numbers show the positions of amino acid residues. Single blue lines in the domains show loss of amino acids. Amino acid sequences (black bold upper-case letters) show newly inserted amino acid fragments. Amino acid sequences following incomplete domain were due to frame-shift reading, resulting in a premature stop (red color) codon.

The surveyor mutation detection assay was performed to detect the mutated individuals among both dead embryos and hatched fry. Digested PCR products revealed that non-edited fish showed one distinct band (482 bp), while the individuals carrying mutated genes showed two or more bands (<482 bp). Gel electrophoresis revealed various banding patterns depending on the type of mutations in the four treatment groups (Fig. 3Aa). Fin clips are the most desirable tissue to assay, as they cause minimal damage to the fish and they regenerate. If fin clips mirror the mutation type found in all tissues, the fish could be sorted based on mutation rates scored in fin clips only without sacrificing the fish. The mutations in every tissue tested, such as barbel, muscle, intestine, and eye, were found to have identical banding patterns with those found in the fin clips (Fig. 3Ab).

All dead embryos from all treatments were mutated, while mutagenesis frequencies in fry among treatment groups MSTN-1, MSTN-2, MSTN-3 and MSTN-Mix were: 88.6%, 100%, 88% and 96.3%, respectively. The PCR amplicons from mutated individuals were cloned and sequenced to confirm and characterize the mutation. The alignment of mutated DNA sequences against the wild type sequence revealed multiple forms of indels caused by the CRISPR/Cas9 system at target sites (Fig. 3C–Fa). Mutated individuals from the MSTN-1 group exhibited two types of deletions, 15 and 36 bases, and a 6-base insertion (Fig. 3Ca). However, Cas9 nuclease cut at the target site, but there was no frame shift because the base deletions and insertion were triplets, resulting in missing and/or additional amino acid (Fig. 3Cb). In the MSTN-2 treatment, only deletions were found (Fig. 3Da); 4, 13, 19, 22, 25, 26, 130 and 207 nucleotides that all should lead to frame shifts (Fig. 3Db). The MSTN-3 group had four types of deletions (2, 8, 9, and 30 bp) and three types of insertions (5, 6 and10 bp) leading to frame shift mutations in two deletion and insertion types and in-frame mutations in the rest (Fig. 3E). The most variable indel mutations (including large deletions) occurred in the MSTN-Mix Group. Exon I of MSTN was almost deleted. While exon I involves 418 bp (Fig. 1B), five long deletion types; 153,165, 238, 240 and 316 bases were deleted between the target sites of the sgRNA(s) (Fig. 3F). Mutation frequencies as well as predicted gene expression from mutated sequences revealed that most forms of insertions and deletions led to frame shift, resulting in a premature stop codon in the transcribed mRNA and a truncated nonfunctional protein product (Fig. 3B–F).

### Evaluation of growth in MSTN mutant fry

**Figure 4:**
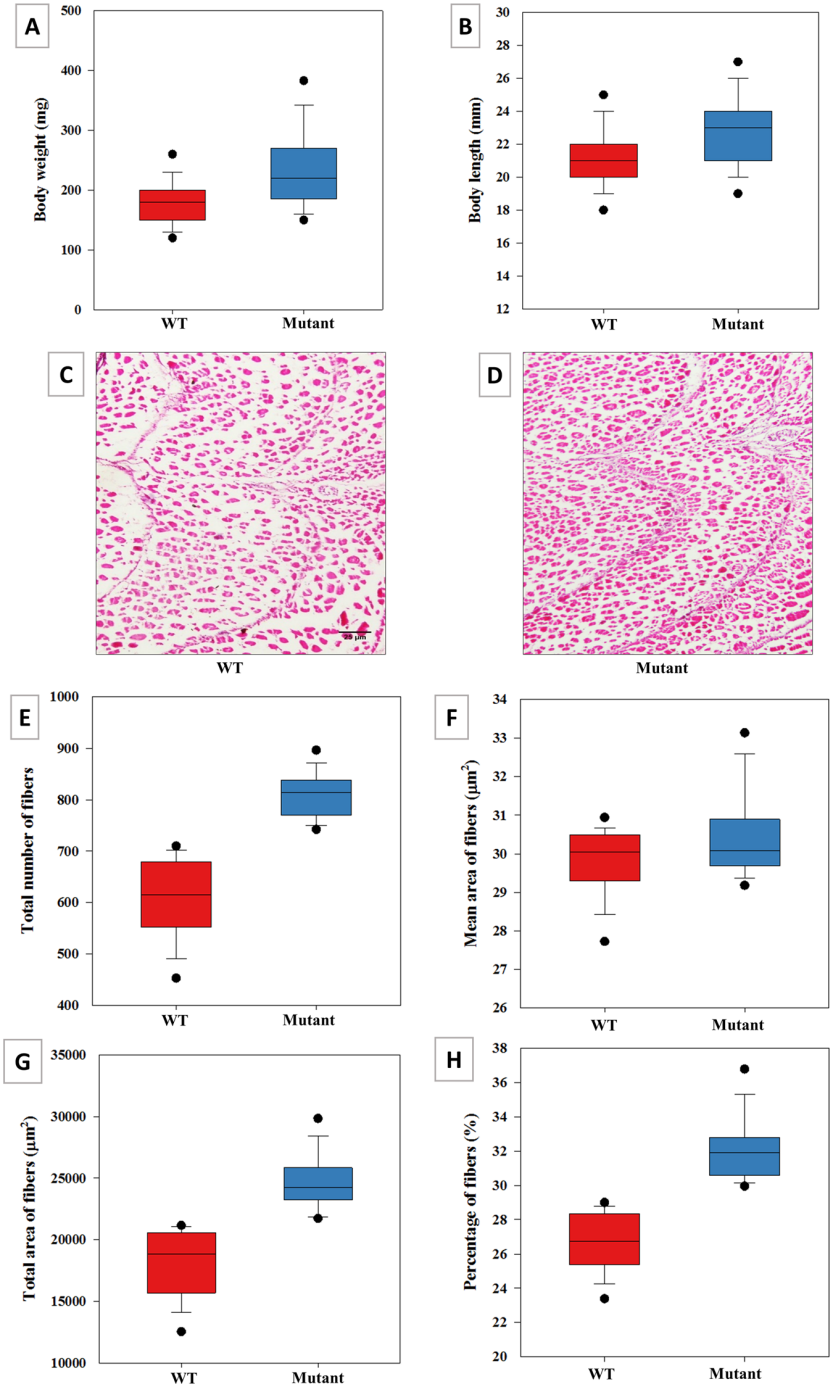
Evaluation of growth in myostatin (MSTN)-mutated one-month-old channel catfish fry. Body weight (**A**) and body length (**B**) of mutant (blue) and wild type (red) (*n* = 330). (**C**,**D**) Representative images of the ventral cross-sectional area of the epaxial muscle of wild-type (WT) (**C**) and mutant (individuals with frame-shift mutation from MSTN-Mix group) (**D**), shown by Hematoxylin and Eosin (H&E) staining. Scale bar in (**C**,**D**): 25 µm. Numbers of muscle fibers (**E**), the average area of the muscle fibers (**F**) and the total area of fibers (**G**) of mutant (blue) and wild-type (red), quantified H&E staining images (see **C**,**D**) with ImageJ. Approximately 32 stained images for each treatment were quantified. Distribution percentage of the fibers (**H**) was calculated as the total area of fibers per cross-sectional area multiplied by 100. Statistical significance was set at *p* < 0.05, and all data were presented as the mean ± standard error (*SEM*).

The average body weight of mutated fry at 40 days post-microinjection was 29.7% larger than wild-types (234.3 ± 6.9 mg vs 180.6 ± 2.77 mg, *n* = 330, Fig. 4A). The mutants also showed 6.6% longer average body length than wild-types (22.7 ± 0.24 mm vs 21.3 ± 0.1 mm, *n* = 330, Fig. 4B). The Shapiro–Wilk normality test revealed that the body weight and body length data were not normally distributed (*W* = 0.95, *p* < 0.0001). Therefore, Mann–Whitney *U*-tests were used to compare differences in body weight and body length between groups. This test revealed that the mutants were significantly heavier (*U* = 5399, *p* < 0.001) than wild-types. The length of mutants was significantly greater than wild-types (*U* = 7633.5, *p* < 0.001).

#### Histological Analysis

The dorsal muscle of mutated channel catfish in Fig. 4D exhibited 33.7% more muscle fibers (809.5 ± 7.9) than wild-types (605.2 ± 13.7), *t*-test: *t*_(49.3)_ = 12.90, *p* < 0.001. The average size of muscle fibers was larger in mutated channel catfish compared to wild-types (30.4 ± 0.2 µm^2^ vs 29.8 ± 0.2 µm^2^), *t*-test: *t*_(62)_ = −2.22, *p* = 0.015 (Fig. 4C–F). Additionally, the average total area of muscle fibers was increased in the mutant (24668.6 ± 394.8 µm^2^) as compared to the wild-type channel catfish (18109.7 ± 467.4 µm^2^), *t*-test: *t*_(62)_ = −10.72, *p* < 0.001. The percentage of muscle fiber area was also higher in the mutants (32 ± 0.3%) than in wild types (26.6 ± 0.3%), *t*-test: *t*_(62)_ = −11.91, *p* < 0.001 (Fig. 4G,H). Altogether, these findings indicate that mutated channel catfish showed hyperplasia as well as hypertrophy.

## Discussion

The sgRNA(s) and Cas9 protein were directly co-injected into one-cell embryos, and gene editing of the channel catfish MSTN gene was successfully accomplished. We utilized CRISPRscan to design the sgRNAs and selected the sgRNAs with the highest scores to target the *I. punctatus* MSTN gene. As CRISPRscan was originally designed based upon the zebrafish (*Danio rerio*) genome^60^, we assume that the phylogenetically close relationship between the genomes of channel catfish and zebrafish^61^ had an important impact on the successful targeting of these designed sgRNAs in channel catfish. All designed sgRNAs worked effectively at their target sites on the MSTN gene with high mutagenesis efficiencies. The mutation rate in teleosts using CRISPR/Cas9 system varies, fluctuating from 2%^55^ to 99%^52^. Although the CRISPR/Cas9 system (Cas9 plasmid) was previously microinjected in channel catfish in our laboratory with a 100% mutation rate^58^, embryo hatch and early fry survival rates were only 10% and 45%, respectively. In the present study, these rates were raised prominently to about 42% for hatching and 90% for early fry survival with high mutation rates (88–100%) (Table 2). Also, the overall mutation rates were higher than those obtained in Atlantic salmon (*Salmo salar*)^56, 62^, Common carp (*Cyprinus carpio*)^59^ and Nile tilapia (*Oreochromis niloticus*)^57^.

To assess the effects of injection and CRISPR/Cas9 system on embryo survival, hatch rate and early fry survival, we included an injected control group as well as a normal control group using injection solution devoid of CRISPR/Cas9 system components. We did not observe any abnormalities pre- or post-hatching. The higher embryo mortality in injected groups compared to the nCTRL group may be due to the microinjection procedures since all embryos were full-siblings, exposed to the same handling stress, and reared using the same environmental conditions.

Oligonucleotides are most active when injected directly into the embryo cell rather than the yolk^63^. However, this injection procedure needs careful orientation of the embryos, and is more time consuming. We co-microinjected Cas9 protein and sgRNA(s) directly into the yolk at the one-cell stage so that it would be transferred to the cytoplasm through cytoplasmic streaming^64, 65^. This approach proved adequate to edit the genome, requiring less time and effort during microinjection with less disruption of embryos, leading to high mutation and survival rates. This approach could explain why the embryo hatch and early fry survival rates were high in this study compared to that of Qin *et al*.^58^ using microinjection of the blastodisc or electroporation. Also, using Cas9 protein instead of its plasmid eliminated the time required for expression of Cas9 plasmid, making genome targeting during the one-cell stage (about 90 minutes in channel catfish) of embryonic development more likely and with high mutation rate.

CRISPR/Cas9 was highly effective as a large percentage of the embryos were mutated within the target sites along the open reading frame, and no mutations were detected nearby and outside the target site. The genome was not examined for off-target mutations. Most mutations should lead to a frame-shift that results in a premature stop codon, early termination in translation, and disrupt the molecular functions of the protein. Fortunately, about two-thirds of the deletions and insertions were frame-shift mutations which most likely caused gene truncations (Fig. 3B–F). Compared to wild-type, the patterns and lengths of amplicon bands revealed with gel electrophoresis differed, depending on the treatment group of the mutated fry. Unsurprisingly, the mutated samples of MSTN-Mix group showed the most variable banding patterns as revealed by gel electrophoresis. In the same manner, the alignment of MSTN-Mix group sequences revealed the most variable types of mutation.

Another important finding was the large deletion in exon I of the MSTN gene resulting from the injection of the three sgRNAs in combination (the MSTN-Mix group). CRISPR/Cas9 system could be used to achieve large genomic deletions, not only in one gene, but also in different chromosomal loci by delivering two pairs or more of sgRNAs together, with Cas9 nuclease targeting different genomic sites simultaneously^51–54^.

Since applied genetics research focuses upon economically important traits, hyper-muscularity after targeting the MSTN gene has been studied in many mammals^44–48^. The average body weights of MSTN-mutant mammals were 15–30% higher than wild-types^46–48^. Regarding MSTN-mutated teleosts, the growth rate enhancement of MSTN mutants was 10–15%^39^ to 39–45%^66, 67^. These results were with the small model species, zebrafish, which does not always translate into the same results in larger fishes. In the present study, we evaluated the phenotypic effects on muscle mass growth in the earliest weeks post-hatching. The mean body weight of mutated fry was increased by 29.7%. The mutant genotypes exhibited hyperplasia (33.7% increase in fiber number) and hypertrophy (2% increase in fiber diameter) of muscle fibers as compared to wild-type, and was the likely explanation of the growth differences between the mutants and wild-types. The number and size of muscle fibers are highly important factors in determining body size in teleosts^68–70^.

This is the first time that Cas9 protein has been directly delivered to edit genes in this biologically and commercially interesting teleost species, channel catfish. We anticipate that this technology will be the principal tool for further molecular and functional studies, especially for channel catfish since its genome is now known^61^. Our study goes beyond vertebrate models (zebrafish and medaka) and addresses the utility of the CRISPR/Cas9 as a tool for generating gene-edited channel catfish, and potentially other aquaculture species, with high efficiency and accompanied with significant phenotypic change. This technology is also especially relevant for teleosts with extended generation times, 2-4 years, like channel catfish, as the extensive mutation in virtually all individuals and especially in all tissues allows solid phenotypic evaluation in the founding generation of channel catfish without having to wait to generate subsequent generations. Further study is still needed to evaluate the carcass composition and meat-quality traits of mutated individuals when they reach commercial food size (400–700 g). Other physiological parameters and immune status should also be considered^39, 71, 72^ and linked to the productivity of these gene-modified lines.

## Materials and Methods

### Design and preparation of sgRNA and CRISPR/Cas9 System

Using the CRISPRscan online tool^60^, three small guide RNAs were customized targeting the *I. punctatus* MSTN gene (GenBank Accession No. AF396747.1). The cloning-free (PCR-based) method to generate sgRNA templates was used; The universal primer containing the sgRNA scaffold as well as ssDNA templates for sgRNAs containing the T7 promoter and the 20-nt gene-specific target sequence without the PAM were manufactured by Invitrogen (Carlsbad, CA) (Table 1). The sgRNAs were generated by T7 run –off as described previously^73, 74^ with some modifications; the universal primer and ssDNA templates were annealed and filled by Platinum™ *Taq* DNA Polymerase (Invitrogen). In RNAse-free environment, the resulting double-stranded DNA served as the template for *in vitro* transcription to generate sgRNA using the Maxiscript T7 Kit (Thermo Fisher Scientific). The obtained sgRNAs were purified using Zymo RNA Clean and Concentrator Kit (Zymo Research, Irvine, CA). The Cas9 protein was from PNA BIO Inc. (Newbury Park, CA) and reconstituted following the manufacturer’s guidelines. Four sets of injection solutions were prepared; three by mixing each individual sgRNA with Cas9 protein separately, and the fourth by combining the three sgRNAs together with Cas9 protein. Phenol red was added to color the sgRNA/Cas9 solutions by mixing sgRNA, Cas9 protein and phenol red in a 1:1:1 ratio. The final concentrations of sgRNA and Cas9 protein were 150–200 ng/µl and 300–350 ng/µl, respectively. The mixtures were then incubated for 10 minutes on ice before loading into the microinjection needle.

### Ethical statement

Channel catfish were obtained from the Fish Genetic Research Unit, School of Fisheries, Aquaculture and Aquatic Sciences at Auburn University, Alabama 36849, USA. The research protocol followed all Standard Operating Procedures (SOP) approved by the Institutional Animal Care and Use Committee (IACUC) of Auburn University.

### Egg collection and sperm preparation

Sexually mature Kansas Random channel catfish females were artificially spawned using luteinizing hormone releasing hormone analog (LHRHa) Reproboost^®^ Implant **(**Center of Marine Biotechnology, Baltimore, MD) at 85 μg/kg body weight to facilitate ovulation, and the eggs were hand-stripped into greased spawning pan. Sexually mature Kansas Random channel catfish males were euthanized and the testes were crushed and macerated into saline (0.9% NaCl) to release sperm and prepare a sperm solution.

### Fertilization, microinjection and embryo incubation

Approximately, 200–300 eggs were transferred to a greased spawning pan. Then, 1–2 mL of the normal sperm solution were added to the eggs and mixed gently. Fresh water was added to the eggs and gently swirled for 30 seconds to activate the sperm and eggs. More fresh water was added and the eggs were allowed to harden for 10–15 min before microinjection. The fertilized eggs were injected according to the procedures for zygote injection developed and modified recently in our laboratory^75^. Briefly, a 1.0 mm OD borosilicate glass capillary was pulled into two needles with a vertical needle puller. A very thin layer of vegetable shortening was applied to a 150 mm clean petri dish. Fifty to one-hundred eggs were transferred from the fertilization pan to the petri dish in a single layer and covered with Holtfreter’s solution (59 mmol NaCl, 0.67 mmol KCl, 2.4 mmol NaHCO_3_, 0.76 mmol CaCl_2_, 1.67 mmol MgSO_4_)^76, 77^. Using a microinjection system from Applied Scientific Instrumentation (Eugene, OR), 50 nanoliters of the mixture were directly injected into the yolk of each fertilized egg. One-cell embryos were injected through 15–90 minutes post-fertilization and just before the beginning of the first cell division^78, 79^. The injected control embryos were injected with the solution devoid of sgRNA/Cas9 mixture. The injected and control embryos were reared in 10-L tubs filled with Holtfreter’s solution containing 10 ppm doxycycline and incubated with continuous aeration at 27 °C for 6–8 days until hatching. Dead embryos were removed and the solution was changed daily. Channel catfish fry then were kept in 5-L containers for one month.

### Mutation Analysis

#### Genomic DNA Extraction

Fin-clip samples were collected from one-month-old fry on ice. Five fry from each treatment were euthanized and samples from barbel, gills, muscle, intestine and eye were collected to study possible mosaicism of the mutations among different tissues. The DNA was extracted using the regular protocol^80^, proteinase K digestion followed by protein precipitation and iso-propanol precipitation of DNA. Quality and quantity of DNA were checked with the Nanodrop 2000 spectrophotometer (Thermo Fisher Scientific).

#### Polymerase Chain Reaction (PCR) and Surveyor Analysis

The primer sets for PCR were designed to cover all possible mutation sites (Fig. 1B). PCR was performed using the Expand High Fidelity^PLUS^ PCR System (Roche). The PCR amplification procedure was as follows: initial denaturation for 3 min at 94 °C; followed by 34 cycles of 94 °C for 30 s, 60 °C for 45 s, and 72 °C for 45 s; and a final elongation at 72 °C for 10 min. The resulting PCR product length was verified in a 1.5% agarose gel. The Surveyor^®^ mutation detection kit for standard gel electrophoresis (Integrated DNA Technologies, Coralville, IA) was used to detect mutations^81, 82^; PCR products were denatured and re-annealed as follows: 95 °C for 10 min; 95 to 85 °C at −2 °C/s; 85 to 35 °C at −0.3 °C/s; cooling to 4 °C; and then Nuclease S was mixed with Enhancer S and MgCl_2_ and added to the PCR products above and incubated at 42 °C for 1 hour. The digested products were separated in a 2% agarose gel and compared to those from a non-edited channel catfish (Fig. 3A).

#### Cloning and Sequencing

To confirm and identify the mutations in each treatment, genomic DNA was obtained from five positive mutated individuals for each treatment and amplified with PCR, and the resulting amplicons were cloned into the TOPO^®^ TA Cloning^®^ Kit for Sequencing (Invitrogen, Carlsbad, CA) and then sent for sequencing to Eurofins Genomics (Louisville, KY). Alignments of nucleotides and amino acid sequences were created and interpreted using T-Coffee tool^83^.

### Histology

To clarify the effect of MSTN knockout on skeletal muscle, we performed histological analysis and statistical quantitative analyses of muscle fibers in skeletal muscle of mutant and wild-type channel catfish. Four each of one-month-old fry from mutant (individuals with frame-shift mutation) and control groups were euthanized with tricaine methane sulfonate (MS-222) (Western Chemical Inc., Ferndale, WA). Subsequently, catfish muscles were dissected, cross sectioned and fixed in 4% paraformaldehyde at room temperature for at least 24 hours, and then dehydrated and paraffin embedded using Tissue Tek II^®^ (Sakura Finetek USA, INC, CA). Serial sections were made at 7 μm thickness using a rotary microtome (American Optical Corporation, Southbridge, MA). The sections were mounted on glass slides and stained with the regressive staining method using Harris hematoxylin (VWR International, PA) and eosin with phloxine (Sigma-Aldrich, Inc., MO). Muscle fibers were counted. Cell numbers were calculated as the number of fibers per cross-sectional muscle area using the “Cell Counter” features of ImageJ program^84^ and used for evaluating fiber size.

### Statistical Analysis

Statistical analysis of microinjected embryo survival, hatching and early fry survival were performed with SPSS 23.0 software (IBM Corporation, Armonk, NY). Dead embryos were collected, recorded and assigned a value representing the time of death (days post-fertilization, dpf). Mortality % was calculated as the number of dead embryos in each treatment divided by the total number of embryos in the same treatment and multiplied by 100. Survival curves for embryos and fry and the time to hatch for all treatment and control groups were compared using Kaplan-Meier test. Pairwise comparisons of mean survival and hatch time were performed using Log Rank (Mantel-Cox) test.

Independent samples *t*-tests were used to compare body weight and body length (growth parameters) between mutant (all positive fish from treatment groups) and non-mutated (all negative fish and controls) depending on the surveyor analysis. Independent samples *t*-tests also were performed to compare between mutants and wild types in terms of muscle fiber density (fibers number and size). The protocol for calculating the *t*-value took into account whether variances were homogeneous^85^. The Shapiro–Wilk test was utilized for analysis of normality of the data. The data that were not normally distributed were analyzed with the Mann–Whitney *U*-test (two-tailed). Analyses were performed with SAS^®^ version 9.4 (SAS Institute, Cary, NC). Statistical significance was set at *p* < 0.05, and all data were presented as the mean ± standard error (*SEM*).

### Data availability statement

All data generated or analysed during this study are included in this published article (and its Supplementary Information file).

## Electronic supplementary material

Supplementary Figure
