# Supplementary material for: Generation of Myostatin Gene-Edited Channel Catfish (Ictalurus punctatus) via Zygote Injection of CRISPR/Cas9 System
Source: Sci Rep. 2017 Aug 4;7:7301. doi: 10.1038/s41598-017-07223-7 (PMC5544710; doi:10.1038/s41598-017-07223-7)
Supplement: Supplementary file 1 — Supplementary Figure [file 41598_2017_7223_MOESM1_ESM.pdf]

Supplementary Information

Generation of *Myostatin* Gene-Edited Channel Catfish (*Ictalurus punctatus*) via Zygote Injection of CRISPR/Cas9 System

Karim Khalil[1, 2,*], Medhat Elayat[2], Elsayed Khalifa [2], Samer Daghash[2], Ahmed Elaswad[1, 4,*], Michael Miller[3], Hisham Abdelrahman[1,5], Zhi Ye[1], Ramjie Odin[1], David Drescher[1], Khoi Vo[1], Kamal Gosh[1], William Bugg[1], Dalton Robinson[1] & Rex Dunham[1,*]

[1]School of Fisheries, Aquaculture and Aquatic Sciences, Auburn University, Auburn, AL, 36849, USA.

[2]Anatomy and Embryology Department, Faculty of Veterinary Medicine, Cairo University, Giza 12211, Egypt.

[3]Harrison School of Pharmacy, Auburn University, Auburn, AL, 36849, USA.

[4]Department of Animal Wealth Development, Faculty of Veterinary Medicine, Suez Canal University, Ismailia 41522, Egypt.

[5]Department of Veterinary Hygiene and Management, Faculty of Veterinary Medicine, Cairo University, Giza 12211, Egypt.

*Correspondence and requests for materials should be addressed to Karim Khalil, Karim.Khalil@vet.cu.edu.eg / Karim11ane@gmail.com, Ahmed Elaswad, ahe0001@auburn.edu, Rex Dunham, dunhara@auburn.edu, School of Fisheries, Aquaculture and Aquatic Sciences, Auburn University, 203 Swingle Hall, Auburn, Alabama 36849, United States. Tel: +1 3348444786; Fax: +1 3348449208.

Figure S1

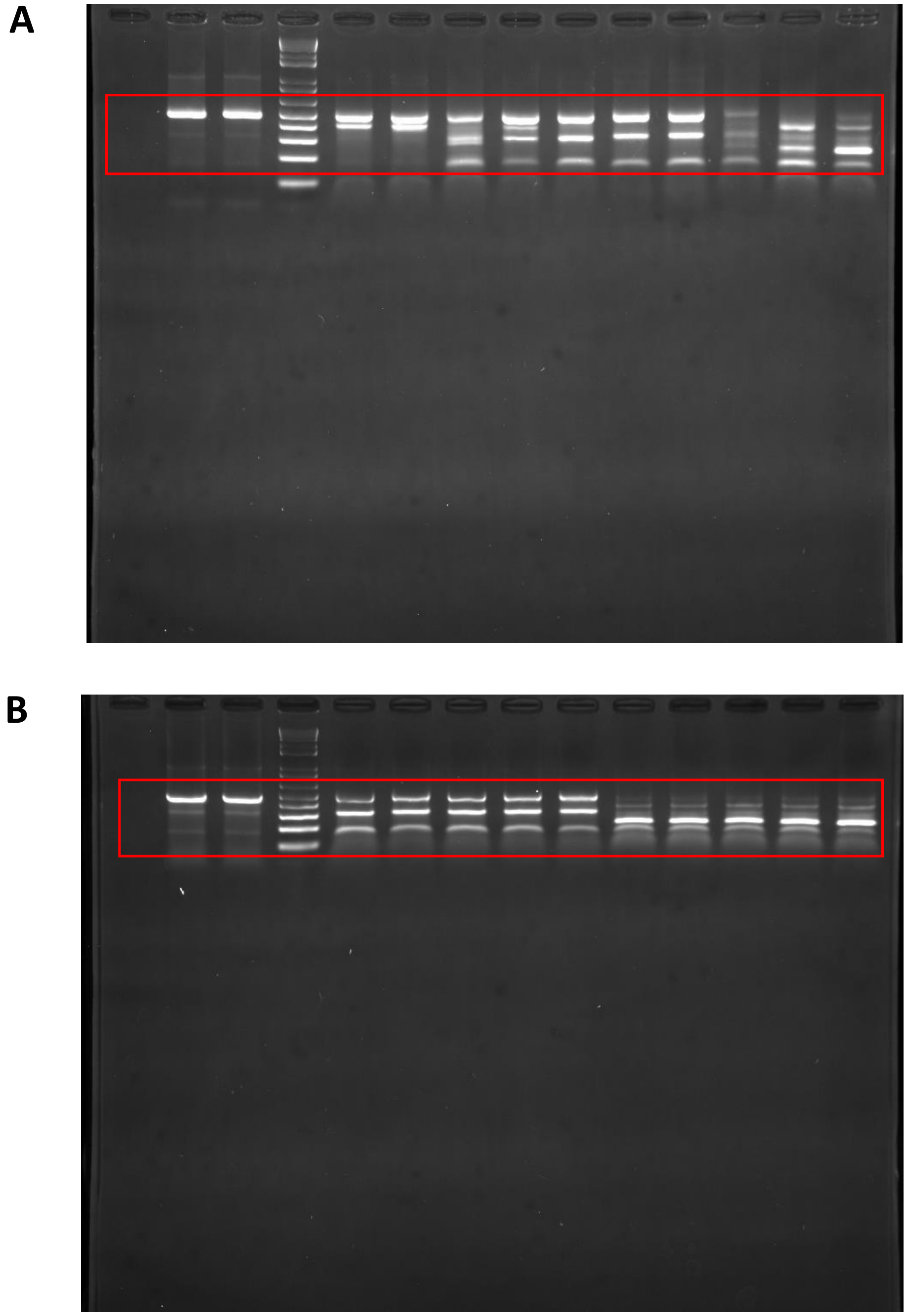

Supplementary Figure S1 Original images of gels.

Results of Surveyor mutation detection in Fig. 3A (A, B) were shown. Red box represent cropped area.
